# Binational climate change vulnerability assessment of migratory birds in the Great Lakes Basins: Tools and impediments

**DOI:** 10.1371/journal.pone.0172668

**Published:** 2017-02-22

**Authors:** Robert S. Rempel, Megan L. Hornseth

**Affiliations:** Ontario Ministry of Natural Resources and Forestry, Centre for Northern Forest Ecosystem Research, Thunder Bay, Ontario, Canada; Auburn University, UNITED STATES

## Abstract

Climate change is a global concern, requiring international strategies to reduce emissions, however, climate change vulnerability assessments are often local in scope with assessment areas restricted to jurisdictional boundaries. In our study we explored tools and impediments to understanding and responding to the effects of climate change on vulnerability of migratory birds from a binational perspective. We apply and assess the utility of a Climate Change Vulnerability Index on 3 focal species using distribution or niche modeling frameworks. We use the distributional forecasts to explore possible changes to jurisdictional conservation responsibilities resulting from shifting distributions for: eastern meadowlark (*Sturnella magna*), wood thrush (*Hylocichla mustelina*), and hooded warbler (*Setophaga citrina*). We found the Climate Change Vulnerability Index to be a well-organized approach to integrating numerous lines of evidence concerning effects of climate change, and provided transparency to the final assessment of vulnerability. Under this framework, we identified that eastern meadowlark and wood thrush are highly vulnerable to climate change, but hooded warbler is less vulnerable. Our study revealed impediments to assessing and modeling vulnerability to climate change from a binational perspective, including gaps in data or modeling for climate exposure parameters. We recommend increased cross-border collaboration to enhance the availability and resources needed to improve vulnerability assessments and development of conservation strategies. We did not find evidence to suggest major shifts in jurisdictional responsibility for the 3 focal species, but results do indicate increasing responsibility for these birds in the Canadian Provinces. These Provinces should consider conservation planning to help ensure a future supply of necessary habitat for these species.

## Introduction

Assessing the vulnerability of species to the effects of climate change is an important approach for conservation agencies that want to develop strategies for mitigating or responding to the effects of environmental change [1, 2]. Climate change vulnerability assessment (CCVA) is an integrated approach to examine effect of exposure to a changing climate and sensitivity of the species to changing conditions [3]. CCVAs are primarily motivated by jurisdictional mandates to conserve biodiversity in the face of climate change. Although climate change is a global stressor, requiring an international effort to reduce global emissions, most conservation and management drivers are local in scope [4]. As a result CCVAs are often restricted to jurisdictional boundaries, and may be inadequate for species conservation where populations range across international borders and conservation strategies require international efforts [5, 6].

**Fig 1:**
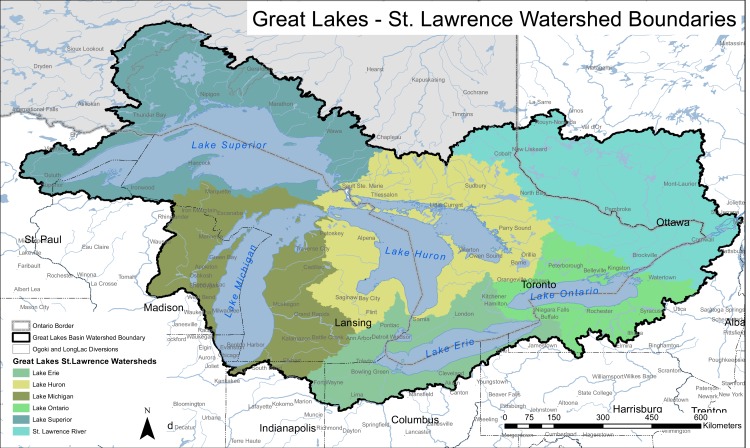
Great Lakes Basin watersheds across Canada and the United States.

The vulnerability of migratory songbirds, whose breeding ranges lie within the Great Lakes Basin, is an interesting example of the problem. Vulnerability assessments are conducted within a defined geographic area, and in this case the assessment area crosses an international boundary separating Canada and United States (US), and is comprised of watersheds falling within two Provinces (Ontario and Quebec) and six States (Minnesota, Wisconsin, Michigan, Ohio, Pennsylvania, and New York) (Fig 1). In addition, the migratory and over-wintering ranges for some of the migratory species found within the basin overlie several countries within North America, Central America, and South America. This binational assessment area creates an interesting, and potentially problematic situation, as the area crosses multiple state, province and international boundaries and associated jurisdictional responsibilities. From a conservation perspective jurisdictional responsibility is in part a function of distribution of the species, and can be defined using a score for responsibility based on proportional distribution among jurisdictions, and a score for concern representing vulnerability and population trend [7]. Climate change can affect the proportional distribution and vulnerability of species, however the magnitude and direction of the impacts are species-specific [8]. All of these factors are important for developing long-term conservation strategies for migratory birds in the Great Lakes Basin.

In addition to this jurisdictional complexity, a diversity of factors influence vulnerability. The effects of climate change on birds within the Great Lakes Basin cross multiple disciplines, as vulnerability is a function of both the fundamental and the realized niche of the species [9]. The realized niche is often modeled using correlative species distribution models (SDMs), which consider the species-specific climate envelope habitat needs (ranging from ecosystem to specific tree distribution). This bioclimatic envelope is further constrained by sensitivity and adaptive capacity of the species, including the interactive effects of dispersal, phenology, competition, genetic introgression, predators, disease, and parasites [9]. From a forecasting perspective models examining global circulation, regionalized temperature and precipitation, tree distribution, landscape and fire disturbance, bird climatic envelope, and meta-population dynamics are all useful tools to support the prediction of how climate change will affect the long-term survival of birds, as well as preparing conservation and management strategies to reduce the chance of extinction [10].

Several challenges to CCVAs have recently been recognized, including omission of a species’ sensitivity and adaptive capacity, focusing on future instead of present climate change threats, and concentration on direct threats of climate change, without identifying indirect threats [11]. However, where the conservation concern is binational in perspective, such as the issue of migratory birds in the Great Lakes Basin, jurisdictional boundaries may further prevent adequate conservation planning because research and management efforts can become fragmented from perspectives on monitoring, analysis, modeling, and policy, and thereby create impediments to vulnerability assessment and development of conservation strategies. Although binational programs such as the Great Lakes Water Quality Agreement [12] are in place to support cooperation and knowledge exchange between nations for aquatic systems (including annexes on climate change impacts, habitat, and science), such agreements do not focus on the non-aquatic, terrestrial components of the Great Lakes basin. The importance of terrestrial systems in the basin for conserving biodiversity has been recognized by some. For example, Nature Conservancy Canada and Ontario Ministry of Natural Resources and Forestry developed a Great Lakes Conservation Blueprint for Terrestrial Biodiversity [13]; however this examines only the Canadian portion of the Great Lakes basin.

Approaching CCVAs from a cross-jurisdictional perspective is still relatively rare, and may be impeded by funding road-blocks, management direction, and an implicit perception that such efforts are unnecessary or only marginally important. Also, it is unlikely that organizations consider the possibility of shifting jurisdictional conservation responsibility for species at risk. The goal of this study is to explore impediments to assessing and responding to the effects of climate change on the vulnerability of birds in a binational context. We do this by testing the policy/research hypothesis that existing assessment and modeling frameworks and tools are sufficient for assessing vulnerability of migratory birds in the Great Lakes Basin of Canada and the US. We assess if sufficient and appropriate data, assessment tools, predictive models and geographic scope of these are in place to enable effective binational CCVA, including the assessment of possible shifting jurisdictional responsibility. To facilitate our evaluation we apply and assess the Climate Change Vulnerability Index (CCVI) as this assessment tool is applicable in both Canada and the US [14, 15]. We evaluate predictive climate change SDMs for three focal bird species, and associated habitat and vegetation response models that were developed for use in either the Canada or the US.

## Methods

### Study area

Our study area included the terrestrial areas of both the US and Canadian watersheds of the Great Lakes Basin totally 516 682km^2^ (Fig 1). The US watersheds extended through the states of Minnesota, Wisconsin, Illinois, Indiana, Michigan, Ohio, Pennsylvania, and New York and the Canadian component included watersheds located in both Ontario and Quebec.

### Selection of focal species

We used a step-wise approach to select three focal species for the assessment that met the joint criteria of being geographically relevant, at greatest risk, having sufficient research on the key issues, and being representative of important habitat types that might be differentially affected by climate change. These were important criteria to test the sufficiency of existing frameworks and levels of binational cooperation for CCVA analysis of migratory species. Throughout the Great Lakes Basin, there are 42 migratory birds listed as ‘at risk’. Of these, 29 occur in Ontario (though not all may be listed as ‘at risk’ in Ontario if they are listed elsewhere). We reviewed the current peer-review literature on the species biology, population and range trends, and climate modeling to determine if the species were appropriate for a CCVA. Seventeen species had sufficient research to complete most of the important questions in the CCVI matrix, including availability of climate-based species distribution models to answer questions related to range expansion or contraction. From these we selected 3 focal species that were present in at least 5 squares (10 km by 10 km UTM grid blocks) of the most recent Ontario Breeding Bird Atlas [16], represented different habitat needs and had the most information available to fill the CCVI matrix, to examine their vulnerability to climate change.

The three selected focal species were eastern meadowlark (*Sturnella magna*), wood thrush (*Hylocichla mustelina*), and hooded warbler (*Setophaga citrina*). Eastern meadowlark is a grassland specialist and is listed as threatened, both provincially under the Endangered Species Act and nationally under the Species at Risk Act (SARA) [17]. Wood thrush a mature deciduous forest specialist, is listed as threatened nationally and special concern provincially [18]. Hooded warbler is a gap phase species preferring mature Carolinian deciduous forest with openings that create a dense understory shrub layer; it is listed as special concern provincially and not at risk nationally [19]. Hooded warbler has undergone recent range expansion in eastern North America over the last 30 years [20]. These 3 species have breeding grounds within the Great Lakes basin, with overwintering grounds in Central America for wood thrush and hooded warbler, and in the southern US portion of its breeding range for eastern meadow lark.

### Assessment of vulnerability

**Fig 2:**
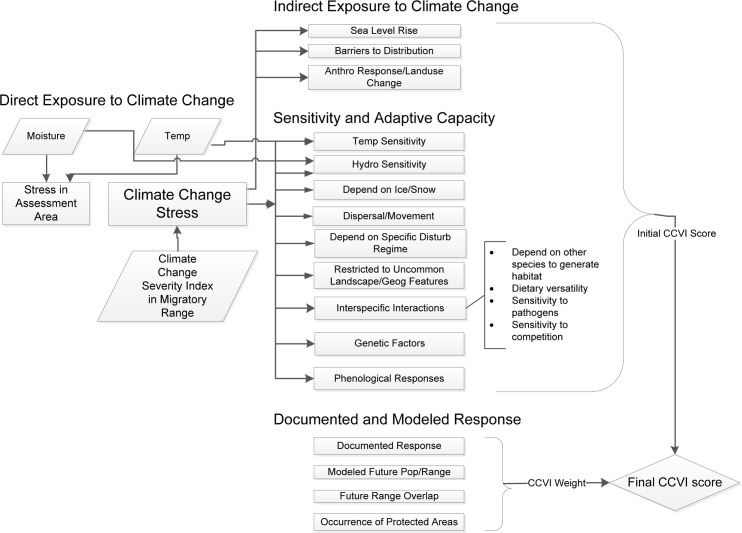
NatureServe’s CCVI based on climate change vulnerability and adaptation strategies for natural communities.

We used the NatureServe CCVI release 3.01 to quantitatively derive a vulnerability index [14, 15]. The Index combines information on exposure and sensitivity to produce a numerical sum, which is then converted to a categorical score (Extremely Vulnerable, Highly Vulnerable, Moderately Vulnerable, Less Vulnerable, and Insufficient Evidence) based on threshold values (Fig 2). If there is an available climate SDM then a combination of the result from the exposure/sensitivity/adaptive capacity section and modeling section is used (but with lower weighting for the modeled section). Details on the scoring mechanism are provided in [21].

Exposure to climate stress was based on predicted magnitude of change to climatic conditions in mid-century (based on mid-level greenhouse gas scenario), and included indices of temperature and moisture severity. Exposure was estimated for the mapped breeding range of the species contained within the Great Lakes Basin, and was calculated (through GIS overlay) as the proportion of the range that falls within each of 6 moisture severity classes. To estimate moisture severity we used the Hamon AET:PET moisture metric [22], which measures the moisture deficit between the 2050 time horizon and the 1961–1990 baseline (ensemble Global Circulation Model (GCM) and medium A1B climate scenario) for the continental US [23]. To estimate severity of temperature change we used data for the entire basin using an ensemble GCM, an equivalent medium greenhouse gas scenario (RCP 4.5), with difference in annual mean temperature based on the difference between the 2050 annual mean and the 1971–2000 baseline [24]. To estimate severity of the exposure to climate change in the overwintering grounds we used the composite Climate Change Exposure Index (CCEI), available at the CCVI site [25]. We estimated indirect exposure to climate change based on predicted impact of land use changes resulting from human responses to climate change (e.g., increased or longer haying season). The index assesses sensitivity of the species to climate change and the adaptive capacity of the species to withstand environmental change [14]. For example, a species with good adaptive capacity can adjust behaviourally or genetically to climate change. The results from the indirect exposure and sensitivity/adaptive capacity sections were then weighted by the scores from the modeled response to climate change section.

We focused on three climate modeling frameworks developed in the US or Canada (Table 1). Although other modeling frameworks exist, such as the framework developed for modeling climate change effects on boreal birds [8], we only considered frameworks that included a substantial portion of the Great Lakes basin. The US Forest Service’s Climate Change Tree Atlas and Bird Atlas project (CC-TABA), used a multistage modeling framework that began with data from the North American Breeding Bird Survey (NA-BBS) and the Forest Inventory and Analysis program [24, 26–28]. From this they developed SDMs for 134 tree species and 147 bird species. The avian models included output from the tree species, which was an important advantage of this modeling framework. Model extent of the tree distribution models was restricted to the US limiting the projection in the Great Lakes Basins to the US watersheds. These statistical models of habitat suitability (DISTRIB) were developed using a machine learning decision tree method (RandomForests) that included climate, elevation, and tree distribution variables. Tree distribution for 134 species was modeled using explanatory variables including climate, soil type, soil characteristics, and landscape variables to predict changes in habitat driven by changing tree distribution [26, 29].The reliability of current SDMs were evaluated as High (>0.5), medium (0.3–0.5) and low (<0.3) [26]. The wood thrush model had high reliability explaining 74% of the deviance, while the eastern meadowlark and hooded warbler models each medium reliability, with 49.2% and 46.6% of deviance was explained, respectively. A 1-km cell-based simulation model (SHIFT) was then used to model possible colonization of suitable new habitat cells (or patches) and the shifting front of species distribution over the next 100 years [29]. Future climate was projected using 3 downscaled GCMs (HADCM3, PCM, and GFDL) and two greenhouse gas emission scenarios (A1FI–where emissions continue to rise without mitigation and B1 –significant conservation effort) [27, 30, 31]. The DIST model estimate predicted exposure to a new climate, while a database of sensitivity and adaptability traits of tree species (MODFACS) help managers develop and assess potential actions.

**Table 1:** Summary of modelling frameworks used to support climate change vulnerability assessment.

Modelling framework	Geographic range	Modeling approach	GCMs, scenarios, and principal data sources
Climate change tree atlas and bird project (CC-TABA) [26–29]. http://www.fs.fed.us/nrs/atlas/	US portion of Great Lakes Basin (Canada excluded)	Integrated modelling framework. SDMs (habitat suitability) developed for trees and birds based on machine learning (Random Forests); distributional changes modeled using cell-based colonization models; outputs assessed in context of tree species adaptability	● Birds: NA-BBS ● Vegetation: Tree database ● Climate: 3 downscaled GCMs (HADCM3, PCM, and GFDL) and two greenhouse gas emission scenarios (A1FI–where emissions continue to rise without mitigation and B1 –significant conservation effort)
Effects of climate change on Quebec biodiversity (vegetation and birds) (CC-QBD) [32]. http://cc-bio.uqar.ca/english/en_atlas.html	● Birds: Most of the Great Lakes; some portions of Minnesota missing ● Trees and Shrubs: US and Quebec portion of the Great Lakes Basin (Ontario excluded)	Partially integrated modelling framework for bird and vegetation SDMs (ecological niche models). SDMs developed from alternative machine learning models (Generalized Additive Models, MaxEnt, and RandomForests) with outputs averaged to estimated expected response.	● Birds: NA-BBS; Quebec Bird Atlas[33] ● Climate: 15 global climate models and 3 projected greenhouse gas emissions scenarios (A2, A1B and B1).
Coupled SDM/meta-population dynamic model for hooded warbler (CC-SDM/MPD) [34]	Breeding range of Hooded warbler (Almost entire Great Lakes Basin)	Hierarchical modeling framework SDMs developed using machine learning (MaxEnt); Spatial distribution using RAMAS GIS; links to meta-population dynamics models; sensitivity analysis tools applied to outputs.	● Hooded warbler: NA-BBS and Ontario Breeding Bird Atlas ● Climate: 4 downscaled GCMs (HADCM3, CCMA, CSIRO, and NIES) using the A2 scenario only.

The Effects of Climate Change on Quebec Biodiversity project (CC-QBD) was initiated by Ouranos, a non-profit group that mediates the relationship between policy and science, and was created as a joint initiative of the Quebec government, Hydro-Quebec, and Environment Canada [32]. This boundary group acts as a catalyst for teamwork. The modeling framework of this group is focused on creating SDMs (they term ecological niche models) to forecast future changes in distribution and abundance of organisms (Table 1). SDMs are based on correlative relationships between primarily climatic variables and the North American Breeding Bird Survey [33] for bird abundance observations, or the Quebec Bird Atlas for presence/absence, but for some species environmental features also included non-climatic variables such as altitude, soil characteristics, and landcover. For bird species, the extent of the modeling area covered almost all of the Great-Lakes Basin, with only a small portion of Minnesota missing. For tree and shrub species, modeling covered the US portion of the Great Lakes Basin, with Ontario excluded and only the Quebec portion included for Canada. Rather than deriving SDMs based on a single modeling approach, this group takes an ensemble approach where they use model-averaging of the various statistical models (e.g., Generalized Additive Models, MaxEnt, and RandomForests) [32]. They use this averaging approach to map shifting climate envelopes. Likewise, they use an ensemble approach to GCMs, where they averaged 70 climate change permutations using 15 global climate models and 3 projected greenhouse gas emissions scenarios (A2, A1B and B1). Mapped species distribution is averaged to provide a perspective on the shifting climate envelope that is not dependent on a single SDM or GCM. Although both bird and vegetation species were modeled, at this point the projections are not well integrated. These climate envelopes applied and averaged across scenarios predict exposure to new climates. Subsequent vulnerability assessments were supported by also considering a species’ life-history traits (using the NatureServe database) to better understand potential sensitivity and adaptability to the new climate.Current SDMs were evaluated using Area Under the Curve (AUC), where AUC scores ≥0.7 indicate good model fit. All models scored ≥0.98.

A third climate change modeling framework coupled an SDM to a meta-population dynamic model (which we term CC-SDM/MPD), but this effort was specific to hooded warbler, and was focused on assessing model uncertainties. A University of Toronto group used this approach to provide projections of habitat suitability over the entire breeding range for hooded warbler, which include almost all of the Great Lakes Basin [34]. The hierarchical modeling framework began with a SDM derived through MaxEnt using the NA-BBS records and point-count locations from the first (1981–1985) and second (2001–2005) Ontario Breeding Bird Atlases and current climate data from the Worldclim database [35]. Climate variables were selected a priori because on biologically relevant factors for migratory birds over the breeding range and were summarized with months associated with the breeding season. The SDMs were project forward using four downscaled GCMs (HADCM3, CCMA, CSIRO, and NIES), as well as an ensemble model, using the A2 scenario only to assess future climate suitability. Additionally, the authors assessed the deviations among GMCs during late-century projections to understand which climate variables were most significant in determining future projections of habitat suitability. Next, the climate SDM was integrated with a metapopulation model using empirical and expert knowledge [36] and dispersal was modelled in RAMAS GIS [37]. A binary map (forest and non-forest) of suitable cover was derived for southern Ontario to map suitable habitat patches of mature forest with openings or gaps greater than 1 km^2^ (Table 1). The relative habitat suitability map based on the SDMs was modified by multiplying it by the binary forest map. RAMAS GIS was then used to identify patches of suitable habitat and link projections to meta-population dynamic models that considered habitat patch occupancy and extinction risk. This model was then used to assess extinction risk under the GCMs as well as the impact of the direct loss of habitat, where patches were randomly removed from the landscape. The influence of various parameters was assessed using the RandomForest decision tree program. The model including all climate variables produced the highest AUC score (0.802).

## Results

From a multinational perspective, the CCVI enabled a basic CCVA for our focal migratory birds, and allowed relevant information to be incorporated from Canada, US and Central America into the CCVA (Table 2). The CCVI considered i) exposure of the species to climate change within the breeding range, ii) indirect climate exposure resulting from human responses to climate change, iii) sensitivity to climate exposure and adaptive capacity, iv) an exposure index for the overwintering grounds, v) modeled distributional changes (or changes in climate envelope) expected under specific climate change scenarios, and vi) documented responses (peer review) to climate change [38]. A concise assessment is provided here, and details of the literature review to complete the CCVI assessment and the full CCVI table is provided in Section A and Table A in S1 File.

**Table 2:** CCVI^1^ scores for eastern meadowlark, wood thrush, and hooded warbler in the Great Lakes Basin (GLB).

Vulnerability Indices	Specific questions	Eastern Meadowlark	Wood Thrush	Hooded Warbler
**Section A: Exposure to Local Climate Change**				
Temperature: Severity (% of GLB)	What percentage of the breeding range will experience a small to large increase in temperature?			
>6.0° F warmer		0	0	0
5.6–6.0° F warmer		0	0	0
5.1–5.5 ° F warmer		10	10	0
4.5–5.0 ° F warmer		80	80	50
3.9–4.4 ° F warmer		10	10	50
<3.9 ° F warmer		0	0	0
Hamon AET:PET Moisture Metric: Severity (% of GLB)	What percentage of the breeding range will experience a small to large increase in drier conditions?			
<-0.119		0	0	0
-0.097–-0.119		0	0	0
-0.074–-0.096		20	20	50
-0.051–-0.073		60	55	50
-0.028–-0.050		20	20	0
>-0.028		0	5	0
Migratory Exposure—Climate Change Exposure Index: Severity (% of GLB)	What percentage of the over-wintering range will experience a small to large increase in temperature?			
>7		85	85	80
6–7		10	10	10
4–5		5	5	10
<4		0	0	0
**Section B: Indirect Exposure to Climate Change**				
2) Distribution to a) Natural Barriers	How will the effect of climate on natural barriers to range shifts (e.g., presence of prairie habitat) influence vulnerability?	Increase		
3) Predicted impact of land use changes resulting from human responses to climate change.	How will landuse change, such as spring farming practices, affect vulnerability?	Increase		
**C. Sensitivity and Adaptive Capacity Factors**				
ii) physiological hydrological niche.	How will changes to a specific hydrologic regime (e.g. prairie soil moisture) affect vulnerability?	Increase/ Somewhat Increase		
c) Dependence on a specific disturbance regime likely to be impacted by climate change.	How will an increased fire rate affect vulnerability?	Increase		
4) Interspecific interactions				
a) Dependence on other species to generate required habitat.	How will climate effects on availability of specific tree species affect vulnerability?	Increase/ Somewhat Increase	Increase	Somewhat increase
b) Dietary versatility (animals only).	For birds with specific diets, how will climate change affect food supply and their vulnerability?		Increase	
e) Sensitivity to pathogens or natural enemies.	How will changes to the prevalence of pathogens that attack specific tree species affect vulnerability?		Increase	Somewhat increase
c) Reproductive system				
6) Phenological response to changing seasonal temperature and precipitation dynamics.	How will a species’ inability to change its breeding arrival dates and behavior affect vulnerability?	Increase/ Somewhat Increase	Increase	Somewhat Increase
**Section D: Documented or Modeled Response to Climate Change**				
2) Modeled future (2050) change in population or range size	If published SDMs predict changes to population size or range size, how will this affect vulnerability?	Increase	Increase	Somewhat increase
3) Overlap of modeled future (2050) range with current range	If overlap of predicted future range and current range changes, how will this affect vulnerability?	Somewhat Increase	Somewhat Increase	Somewhat increase
4) Occurrence of protected areas in modeled future (2050) distribution	How will the presence of parks and refuges in the predicted future range affect vulnerability?		Neutral	
**Vulnerability to Climate Change Scores**				
Climate Change Vulnerability Index (CCVI)		Highly Vulnerable	Highly Vulnerable	Less Vulnerable
Confidence in Vulnerability Score		Very High	Very High	Very High
Climate Exposure in Migratory Range		High	High	High
**Conservation Concern**				
COSEWIC (National—Canada)		Threatened	Threatened	Not at Risk
SARA (Ontario)		Threatened	Special Concern	Special Concern
NatureServe G-rank (Global)		Secure	Secure	Secure

1. Detailed explanation of variables provided in the CCVI spreadsheet [38]. Only values that were scored we included in this table.

Some of the readily assessable data required to populate the CCVI table was restricted to specific jurisdictions (Table 2). For example, to estimate moisture severity we used the Hamon AET:PET moisture metric [22], which measures the moisture deficit between the 2050 time horizon and the 1961–1990 baseline (ensemble GCM and medium A1B climate scenario) for the continental US [23]. AET:PET values were taken from the packaged climate summaries available on the CCVI site, and produced by the Climate Wizard development team [25, 39]. For the Canadian portion of the basin, however, we had to extrapolate values to estimate approximate moisture deficit values. Approximately 53% of the basin is in Canada, and the amount of extrapolation required differs among species. Approximately 25% of the hooded warbler breeding range required extrapolated moisture change values, while close to 50% of meadow lark and wood thrush required extrapolated values (and encompassed a much greater area than the hooded warbler range). Patterns of moisture change were fairly homogenous on the US side, so we assumed that they would be equally homogenous on the Canadian side. The extrapolation was not precise and is subject to unquantifiable error, but provides an approximate estimate of how moisture conditions will change in the area.

Predictive temperature data required for the suggested GCMs and scenarios for CCVI were available in GIS for only for the US portion of the basin, although the issue here may be related to technical problems with web-based tools to generate data for a specific geographic area rather than management decisions to restrict extent of data. To resolve this issue, we used predictive CC data that included both the US and Canadian portions of the basin, and that was based on a mid-century (2050s), ensemble GCM and a medium emission scenario (RCP 4.5) and is roughly equivalent to the CCVI suggested A1B scenario. An additional consideration was GIS conversion of Celsius to Fahrenheit, as the North American predictive temperature data we used was mapped in Celsius while the CCVI requires estimates in Fahrenheit. We had no issues estimating the severity of the exposure to climate change in the southern US and Central American overwintering grounds as all the necessary data was available at the CCVI site [25].

**Fig 3:**
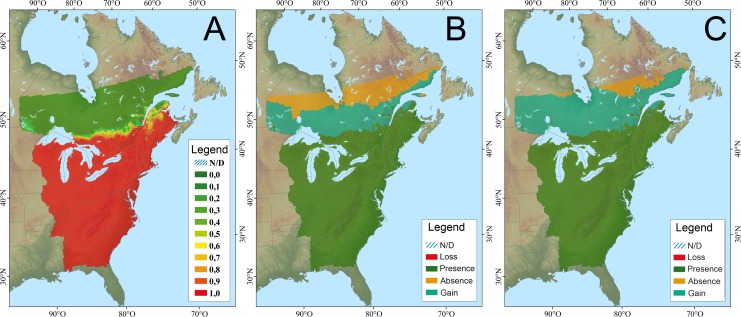
Climate niche model for eastern meadowlark; A. modeled current distribution (probabilities of occurrence; 1961–1990); B. potential distribution change (2041–2070); C. potential distribution change (2071–2100) (from CC-QBD–Berteaux et al. [40]).

The basic modeling frameworks, including species distribution (SDM) and vegetation models varied among both jurisdictions and species (Table 1). Geographically, SDMs were generally available for the entire breeding range, while more specific vegetation response models were largely restricted to the US portion of the basin. For eastern meadowlark both the Canadian-based CC-QBD and US-based CC-TABA modeling frameworks were used to evaluate changing climate envelopes and habitat. For the 2041–2070 time horizon the CC-QBD SDM models predicted a net gain in habitat area resulting in a northward expansion of 307 km from 1990 to 2070 (Fig 3), and 465 km by 2071–2100 horizon [32]. However, the grassland habitats that eastern meadowlarks inhabit are unlikely to move northward as quickly, as the northern region lies on the boreal shield, where grasslands are rare. In contrast, the CC-TABA models predicted a decrease in abundance in the western part of the eastern meadowlark range by 2100 and do not predict any movement in the core of the range or northern expansion [27, 28]. Model predictions encompassed the eastern US, but did not include any predictions north of the border due to a lack of climate-vegetation modeling in Canada. The overall CCVI score for meadowlark was highly vulnerable (Table 2).

**Fig 4:**
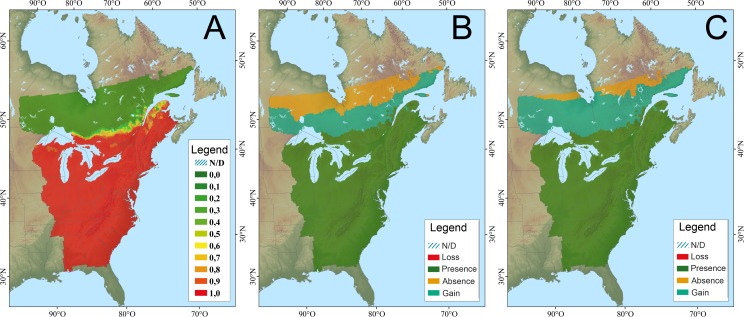
Climate niche model for wood thrush; A. modeled current distribution (probabilities of occurrence; 1961–1990); B. potential distribution change (2041–2070); C. potential distribution change (2071–2100), (from CC-QBD–Berteaux et al. [40]).

Wood thrushes are mature forest species found in mixed wood and deciduous forests, often preferring previously disturbed sites [41]. They nest in interior edges [42], selecting deciduous trees such as American beech (*Fagus grandifolia*), American elm, and red maple (*Acer rubrum*) for nesting [43]. For wood thrush the CC-QBD model predicts a range expansion with an increase in habitat area by 25.5%, a northward range expansion of 28 km/decade for an overall range extension of 304 km by 2041–2070 (Fig 4) with an additional 119 km by 2071–2100 [32]. However, the deciduous trees that wood thrushes select for nesting are unlikely to move northward as quickly. The northern range limit lies on the boreal shield, where nesting tree species such as American elm, American beech and red maple are rare. In contrast, the US-specific CC-TABA CC-vegetation models predicted a decrease in abundance in the western and southern parts of the wood thrush range [28]. The top predictors in this model were red maple distribution, annual precipitation, American beech distribution, American elm distribution, and the mean difference between July and January temperatures [27]. Given the jurisdictional boundary of the modeling, it is difficult to extrapolate the changes at the northern range limit into Canada; however, current research suggests that American beech may decline throughout its range across eastern US, while American elm may increase within the northern extent of its range across the northern US [31]. The overall CCVI score was highly vulnerable (Table 2) for wood thrush.

**Fig 5:**
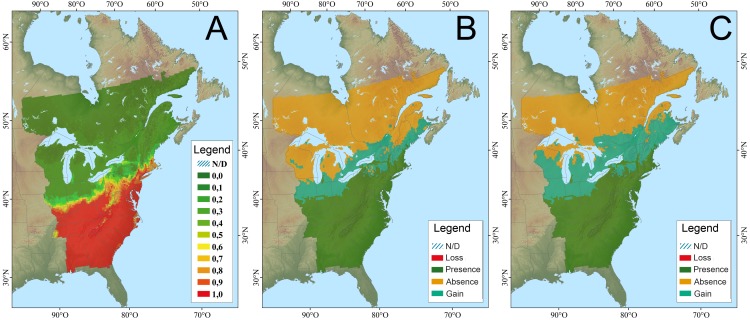
Climate niche model for hooded warbler; A. modeled current distribution (probabilities of occurrence; 1961–1990); B. potential distribution change (2041–2070); C. potential distribution change (2071–2100), (from CC-QBD–Berteaux et al. [40]).

Hooded warblers breed from southern Ontario east to Rhode Island, south through northern Florida, and across the Gulf Coast through northeastern Texas [44] (Fig 5). These birds breed in mature forest that has gaps or openings where early successional vegetation grows. Within the Great Lakes, hooded warblers are found only in the basins of Lake Michigan, Lake Erie, and Lake Ontario. Hooded warblers occupy deciduous forest stands dominated by maple, American beech, and oak (*Quercus* spp.) [44]. For hooded warbler all three modeling frameworks predicted northern expansion in range in relation to different climate scenarios. The Canadian CC-QBD models predicted a range expansion with an increase in habitat area by 28.6%, a northward range expansion of 34 km/decade for an overall range expansion of 378 km [32] by 2041–2070, and a further 156 km expansion by 2071–2100 (Fig 5). The US CC-TABA model predicts a decrease in abundance in the southern part of the hooded warbler range and an increase in the northern US part of their range through the eastern seaboard as well as northern Minnesota, northern Wisconsin, and Michigan, including the upper peninsula [27].

For hooded warbler only, the coupled species distribution—metapopulation dynamics model (CC-SDM/MPD) offered additional insights related to uncertainty based on choice of GCM and demographic parameter uncertainty. This modeling effort was applied to the entire Great Lakes Basin, so extrapolation of results across jurisdictional boundaries was not necessary. The researchers found that interpretations of vulnerability were influenced in part by the particular GCM selected for modeling climate change, but that vulnerability was also strongly affected by habitat loss. These results point to concerns with using alternative GCMs or emission scenarios to estimate temperature and to the inadequacy of using only climate based predictions and not accounting for habitat change. The overall CCVI score was less vulnerable (**Table 2**) for hooded warbler.

## Discussion

We utilized three modeling frameworks, in conjunction with NatureServe’s CCVI, to assess the whether current tools and geographic extent of data are sufficient to conduct CCVAs for migratory birds in the Great Lakes Basin. Our study indicated current modeling efforts are in some cases restricted by jurisdictional boundaries; this impedes vulnerability assessments by necessitating extrapolations to complete the CCVI. For species that are already experiencing population and habitat declines, conservation planning and research efforts should include a coordinated binational approach to ensure that conservation strategies are effective and suitable for those species that are most vulnerable to climate change.

We discovered several impediments to assessing the impacts of climate change on avian species and jurisdictional shifts, including border-related data gaps in climate exposure parameters, as well as modeling frameworks and a lack of examination of critical habitat factors. Despite these impediments, we were able to use the CCVI to identify that eastern meadowlark and wood thrush are highly vulnerable to climate change, but hooded warbler is less vulnerable. Our results support previous research that highlights the importance of understanding the individuality of species’ vulnerability to climate change to appropriately inform conservation efforts [8, 45, 46]. Modeling frameworks that integrate critical habitat factors extend the concept of the climatic envelope into a bioclimatic envelope of necessary conditions, and consequently better define the fundamental niche. All three modeling frameworks included SDMs (or climate envelopes) and were based on current climatic correlations with current species distribution.

Two of the modeling frameworks, CC-TABA and CC-QBD, provided online interactive mapping functions, and this greatly aided the ability to explore changes in predicted distribution and abundance. However, differences in model output, including base GCMS and categorization of predicted presence and absence made direct comparison difficult. CC-TABA provided a richer set of interactive maps overall, including predicted tree distribution, but mapping was restricted to the US portion of the basin.

While bird SDMs are a valuable first step [8, 32, 34], they do not explicitly consider other critical habitat factors, such as distribution of edaphic conditions required to support habitat producing species (e.g., specific tree species such as Beech or specific ecosystem types such as tallgrass prairies). For example, the CC-TABA model provided predicted distributional information for tree habitat species, and thus contributed knowledge on the predicted future availability of critical habitat. A risk of not including the biotic habitat component is an overly optimistic view of how the species could respond to a shifting climatic regime. The three modeling frameworks we used all included biotic habitat components, but these habitat components often did not extend into the Canadian/Ontario component of the Great Lakes Basin, so generalizations or spatial extrapolations had to be made for the assessment. The SDM components for birds were always based on NA-BBS data, an easily accessible binational dataset on bird distribution, but similar data (or modeling effort) was not available for tree distribution and other biotic factors. As a result, the climate envelope and habitat envelope were not well integrated into a single bioclimatic envelope. Iverson et al. [29] provide a useful review of opportunities and lessons for integrating habitat, disturbance and life-history traits into these distributional models.

One of the modeling frameworks (CC-SDM/MPD) coupled a SDM to a meta-population dynamic model, and we found this particularly interesting [34]. This model was used to assess landscape and habitat level responses specific to hooded warbler, and thereby added a dimension of realism to the CCVA by better estimating the realized niche response. While intensive to produce, process-based models may better estimate responses to future conditions that exist outside the domain of measured current and historical responses to climate change factors [9]. This model showed that habitat loss, as modeled by random patch removal, had a significant impact on extinction risk for this species.

NatureServe’s index explicitly considers factors such as climate exposure, adaptive capacity relevant to migratory birds, and modeled species distribution changes. However, the application of the CCVI required careful consideration of each factor as it may be entered in several places. For example, northern expansion of eastern meadowlark is restricted in some areas due the presence of boreal shield, which in turn is a barrier to expansion of prairie grasslands. This could be considered under dispersal and movement (of grasslands), dependence on other (grassland) species, disturbance regime (to maintain grasslands), distribution relative to natural barriers (boreal shield), and restriction to uncommon geological features or derivatives (grassland edaphic conditions). We decided this was an indirect factor (as presence of boreal shield is itself uninfluenced by climate), and that the factor was a barrier to climate-induced range expansion rather than a modifier expressing sensitivity or adaptive capacity of the species. Of the diverse group of climate related responses we discovered through our literature review for the 3 focal species, no factor was left unaddressed by the CCVI. We should note, however, that the environmental effect of climate change on the breeding grounds, phenology, and habitat can be quite different than those on the wintering grounds [2].The index helps in application of critical thought to the vulnerability review process, and with careful application, we would expect results to be consistent and repeatable among different reviewers.

Our study revealed impediments to assessing and modeling vulnerability to climate change from a binational perspective for the whole of the Great Lakes Basin. For almost every climate exposure parameter considered there were gaps in data or modeling. In some cases models did not exist for Canada (e.g., Hamon moisture metric[22]), and in other cases data was difficult to access because of broken web-links or issues with automated mapping. Best guess extrapolations were used where data was lacking in the Canadian watersheds. Improving ClimateWizard’s packaged climate data [39] to enable easy extraction of Canada and Central American GIS data would eliminate this impediment. Cross-border conservation efforts can be hindered by cutting model outputs at the international border. In general, we recommend increased cross-border collaboration, particularly in the Great Lakes Basin, to enhance the availability and resources needed to improve vulnerability assessments and development of conservation planning and monitoring strategies.

The focal species we selected are all within the northern periphery of their geographic ranges, where habitat amount and quality is lower than in the core of their range [47]. The jurisdictional responsibility for Canada is currently very low (0–1) for these species [48], but our vulnerability assessment suggests a future increase in responsibility for the Canadian Provinces, perhaps to levels 2–3 based on the priority setting tool [7]. Canadian Provinces should consider conservation planning for using these and other focal species to help ensure supply of future habitat conditions that are suitable for these species. This would likely involve collaboration among resource agencies (e.g., Ontario Ministry of Natural Resources and Forestry, Canadian Wildlife Service, and US Fish and Wildlife Service) from both nations.

## Supporting information

S1 File Supporting Information. Section A: Review of climate change literature and modeling results for eastern meadowlark (*Sturnella magna*), wood thrush (*Hylocichla mustelina*), and hooded warbler (*Setophaga citrina*).
Table A: CCVI scores for eastern meadowlark, wood thrush, and hooded warbler in the Great Lakes Basin (GLB).
(DOCX)
