# Supplementary material for: Binational climate change vulnerability assessment of migratory birds in the Great Lakes Basins: Tools and impediments
Source: PLoS One. 2017 Feb 22;12(2):e0172668. doi: 10.1371/journal.pone.0172668 (PMC5321439; doi:10.1371/journal.pone.0172668)
Supplement: S1 File — Section A: Review of climate change literature and modeling results for eastern meadowlark (Sturnella magna), wood thrush (Hylocichla mustelina), and hooded warbler (Setophaga citrina). Table A: CCVI scores for eastern meadowlark, wood thrush, and hooded warbler in the Great Lakes Basin (GLB). (DOCX) [file pone.0172668.s001.docx]

## S1. Supporting Information.

## Section A. Review of climate change literature and modeling results for eastern meadowlark (*Sturnella magna*), wood thrush (*Hylocichla mustelina*), and hooded warbler (*Setophaga citrina*).

# Eastern meadowlark

**Exposure to Climate Change**

**Expected climate change in breeding and overwintering ranges (A)**

Eastern meadowlarks (*Sturnella magna*) are a short-distance migrant and can overwinter through much of their southern range; however, populations at northern range limits migrate where snow and ice restrict foraging. Banding data has documented some birds migrating over 1,000 km to wintering grounds in southern states ([1](#_ENREF_1)). In the Great Lakes Basin portion of the eastern meadowlarks breeding grounds, by 2050, the average temperature is expected to rise between 2.2 – 2.7°C. Drought conditions will increase, with a Hamon AET:PET ([2](#_ENREF_2)) moisture index ranging from -0.073 – 0.096. Within overwintering grounds in the southern US, the Climate Change Exposure Index (CCEI) is largely > 7, indicating exposure to both elevated temperature and drought conditions.

**Distribution relative to natural barriers (B.2.a)**

The grassland habitats that eastern meadowlarks depend on are unlikely to disperse northward quickly, as the more northern regions lie on the boreal shield, where grasslands are rare (see section D below). So although the surficial geology and soil conditions required for grasslands are common in the southern portion of the range, portions of the area of northern expansion do not have these edaphic conditions that contribute to the niche of the species, and would consequently restrict northern expansion. For this reason, the Climate Change Vulnerability Index (CCVI) (B.2.a) was scored “Increase”.

**Predicted impact of land use changes resulting from human responses to climate change (B.3)**

Habitat loss and fragmentation are a concern for conservation efforts regarding eastern meadowlarks due to the natural succession of abandoned agricultural lands to forests, expanding urbanization, and more intensive agricultural practices in southwestern and eastern Ontario ([3](#_ENREF_3)). A longer growing season, as a result of climate change, may increase the suitability of grasslands for agriculture, increasing habitat loss for eastern meadowlarks. In these areas, nesting success is related to the timing of the first cut of hay ([4](#_ENREF_4)). If hay is harvestable at an earlier date, this may negatively affect meadowlarks, especially if they are slower to adapt to changes in phenology. Although eastern meadowlarks are frequent double-brooders and are capable of commencing a second or third nest when previous attempts fail, the overall impact of subsequent nests on the population is low due to poor survivorship, indicating that they may have reduced capacity to adapt to a changing climate ([5](#_ENREF_5)). CCVI (B.3) was scored “Increase”.

**Sensitivity and Adaptive Capability**

**Physiological hydrological niche (C.2b.ii)**

As a grassland specialist, eastern meadowlarks are sensitive to changes in precipitation and moisture regimes. Increased precipitation can improve grassland productivity ([6](#_ENREF_6)). This increase in productivity may improve nesting success ([4](#_ENREF_4)). Climate change is predicted to increase the number of frost-free days and increase minimum temperatures, resulting in an increased growing season ([7](#_ENREF_7)). However, future climate change could lead to highly variable precipitation and more extreme events, including extensive periods of drought and flooding. Eastern meadowlarks arrive on breeding grounds earlier in warmer, wetter springs ([1](#_ENREF_1)). CCVI (C.2b.ii) was scored “Increase” and “Somewhat Increase”.

**Dependence on other species to generate required habitat (C.4.a)**

Eastern meadowlarks are grassland specialists and ground-nesters that naturally depend upon short and tall grasslands as habitat ([8](#_ENREF_8)), but are also found in pastures, hayfields, and croplands ([1](#_ENREF_1)). They select areas with tall grass (25-50cm), a high proportion of grass cover (>80%), abundant litter layer, low shrub cover (<5%), moderate to high forb density, and minimal bare ground ([9-12](#_ENREF_9)). CCVI (C.4.a) was scored “Increase” and “Somewhat Increase”.

**Dependence on a specific disturbance regime likely to be impacted by climate change (C.2.c)**

Recent research has shown that climate change could lead to changes in the prairie-forest border within central North America. This would lead to forested areas being converted to savannas or grasslands by the end of the 21st century due to a predicted reduction in precipitation, more frequent natural disturbances such as droughts, fires and windstorms, and invasions of native and non-native species such as earthworms and white-tailed deer (*Odocoileus virginianus)* ([13](#_ENREF_13)). If this occurs, there could be an increase in grassland habitat suitable for eastern meadowlark within the Great Lakes Basin. CCVI (C.2.c) was scored “Increase”.

**Dietary versatility (C.4.b)**

Eastern meadowlarks are ground-feeding insectivores. They feed by searching through grass as well as probing the soil ([1](#_ENREF_1)). Their diet is mainly composed of Coleoptera (beetles), Orthoptera (grasshoppers and crickets), and Lepidoptera (butterflies and moths), with less than 5% of their diet coming from seeds or vegetation ([12](#_ENREF_12)). Crickets and grasshoppers comprise 26% of their diet throughout the year; however, this increases to 72% in August ([1](#_ENREF_1)). In spring, meadowlarks favour caterpillars, cutworms, and grubs. CCVI (C.4.b) was scored “Somewhat Increase”.

**Phenological response (C.6)**

In the migratory part of their range, birds arrive from early January to late May and commence their fall migration to the wintering grounds between early August to late December ([1](#_ENREF_1)). [Wilson (14](#_ENREF_14)) showed that arrival dates for eastern meadowlark are negatively correlated with the North American Oscillation index, which has been increasing for the last 40 to 50 years ([15](#_ENREF_15)), so birds are more likely to arrive earlier in warmer, wetter springs than in colder, dryer ones ([14](#_ENREF_14)). As a short distance migrant, eastern meadowlarks may have a greater capacity to respond to environmental cues than a long distance migrant, where environmental cues on the wintering grounds could be quite different than those on the breeding grounds ([5](#_ENREF_5)). CCVI (C.6) was scored “Increase” and “Somewhat Increase”.

**Modeled Response of Range Distribution**

**Modeled future (2050) change in population or range size (D.2)**

Both the Effects of Climate Change on Quebec Biodiversity Project (CC-QBD) and the US Forest Service’s Climate Change Tree Atlas and Bird Atlas project (CC-TABA) modeling frameworks were used to evaluate changing climate envelopes. For the 2041-2070 time horizon the CC-QBD models predicted a net gain in habitat area by 28.6%, and that range limits would shift northwards at a rate of 28 km/decade for a total shift of 307 km from 1990 to 2070. During the 2071-2100 horizon, the model predicted habitat area to continue to increase by an additional 18.1%, continued northward range expansion at 42 km/decade to an additional 158 km for a total increase of 465 km, relative to the 1961-1990 modeled distribution ([16](#_ENREF_16)). However, the grassland habitats that eastern meadowlarks inhabit are unlikely to move northward as quickly, as the region lies on the boreal shield, where grasslands are rare.

In contrast, the CC-TABA models predicted a decrease in abundance in the western part of the eastern meadowlark range and limited northward movement. The top predictors in this model were annual precipitation, the mean difference between July and January temperatures, mean January temperature, the abundance of red maple (*Acer rubrum*), and minimum elevation ([17](#_ENREF_17)). This model covered the eastern United States, but does not include any predictions north of the border due to a lack of climate-vegetation modeling in Canada.

The CC-QBD models from [Berteaux, Blois (16](#_ENREF_16)) suggest that the climate envelope for eastern meadowlarks is moving northwards. In contrast, the CC-TABA climate-vegetation modeling does not predict any movement in the core of the range or northern expansion ([17](#_ENREF_17), [18](#_ENREF_18)). The CCVI (D.2) was scored “Increase” and D.3 and D.4 “Somewhat Increase”.

**Additional conservation concerns (not directly related to climate change)**

Eastern meadowlarks have experienced widespread declines over the last 40 years, by 72% ([19](#_ENREF_19)). The annual abundance index is declining, as determined by the annual Breeding Bird Survey across North America (survey-wide) and within Ontario. Recent population trends from the breeding bird survey estimate an annual decline of 3.1% in Ontario ([20](#_ENREF_20)). Declines vary throughout the Ontario portion of the range with higher declines in Southern Ontario and slightly lower declines in eastern Ontario and around Sault Ste. Marie. Overall, the population size is estimated to be approximately 250,000 individuals in Canada ([21](#_ENREF_21)), with the Ontario population estimated at 150,000 ([3](#_ENREF_3)). This species is listed as ‘threatened’, both in Ontario and under the Endangered Species Act, and nationally under the Species at Risk Act ([22](#_ENREF_22)).

# Wood Thrush

**Exposure to Climate Change**

**Expected climate change in breeding and overwintering ranges (A)**

The breeding range of wood thrushes (*Hylocichla mustelina*) extends across eastern North America from northern Minnesota, central Ontario and southern Quebec, east to Nova Scotia, south to northern Florida and the Gulf Coast ([23](#_ENREF_23)). Wood thrushes occur across the entire Great Lakes Basin, with the exception of the northwestern portion of the Lake Superior Basin. Wood thrushes’ winter range extends from southern Mexico along the Atlantic and Pacific coasts through western Panama. Within the portion of the wood thrush breeding grounds contained in the Great Lakes Basins, by 2050, the average temperature is generally expected to rise between 2.5 – 2.7°C. Drought conditions will increase, with an AET:PET moisture index ranging from -0.028 – 0.096. Within the Mexican and Central American overwintering grounds, the CCEI index is largely > 7, indicating exposure to both elevated temperature and drought conditions.

**Sensitivity and Adaptive Capability**

**Dependence on other species to generate required habitat (C.4.a)**

Wood thrushes are mature forest species found in mixed wood and deciduous forests, often preferring previously disturbed sites (e.g. small scale logging and ice storm damage) ([24](#_ENREF_24)). They prefer closed-canopy, mid-successional, moist hardwood forests ([25](#_ENREF_25)). Wood thrushes nest in interior edges ([26](#_ENREF_26)), selecting trees such as American beech (*Fagus grandifolia*), red maple, black gum (*Nyssa sylvatica*), eastern hemlock (*Tsuga canadensis*), flowering dogwood (*Cornus florida*), American hornbeam (*Carpinus caroliniana*), oaks (*Quercus* spp.), and pines (*Pinus* spp.) ([23](#_ENREF_23)). Specifically, wood thrushes select nesting sites with diverse deciduous tree species, closed canopy (>70%), moderate sub-canopy, open forest floor, and moist soil ([23](#_ENREF_23), [26](#_ENREF_26), [27](#_ENREF_27)).

The species of deciduous trees that wood thrushes select for nesting are unlikely to move northward as quickly ([16](#_ENREF_16)). The northern range limit lies on the boreal shield, where nesting tree species such as American elm (*Ulmus Americana*), American beech and red maple are rare. Consequently we scored CCVI (C.4.a) as “Increase”.

**Dietary versatility (C.4.b)**

Wood thrushes are omnivorous, feeding primarily on soil invertebrates as well as fruit. Additionally, they may feed on snails and small salamanders ([23](#_ENREF_23)). Of invertebrates, major food items include Coleoptera (in 38.0% of emetic samples), Diptera (18.5%), Hymenoptera (17.3%), and Lepitoptera (11.9%), feeding on both larval and adult stages of each order ([28](#_ENREF_28)). Additionally, they feed on fruits of elderberry (*Sambucus canadensis*), jack-in-the-pulpit (*Arisaema triphyllum*), and blueberry (*Vaccinium* sp.), when available ([23](#_ENREF_23)). CCVI (C.4.b) was scored “Increase”.

**Sensitivity to pathogens or natural enemies (C.4.e)**

Two of the main nesting tree species for wood thrush are American beech and American elm. Beech trees are susceptible to Beech Bark Disease (BBD) and elms are susceptible to Dutch Elm Disease. BBD arises from interactions between an invasive scale insect and a fungus, resulting in tree death within 3-6 years of infection ([29](#_ENREF_29)). There is a correlation between climate and scale insect populations; where minimum daytime winter temperatures of -34°C or colder in severe years result in a scale insect population dieback ([29](#_ENREF_29)). BBD has been present in Ontario for the last 20 years, however, the range has expanded from 2004-2012 ([30](#_ENREF_30)). Future climate conditions may favour the northward spread of both the scale insect and the fungus that result in BBD, which could increase the prevalence of the disease and recurring outbreaks ([30](#_ENREF_30)). Dutch Elm disease has infected and killed most of the elm trees in southern Ontario and continues to spread northwards. Loss of these two tree species would have a negative effect on wood thrushes. CCVI (C.4.e) was scored “Increase”.

**Phenological response (C.6)**

Wood thrushes migrate across or around the Gulf of Mexico from their wintering range to their breeding range, covering 4,600 km in 13 to 15 days ([31](#_ENREF_31)). Spring migration typically occurs between mid-April to late May, where arrival dates are dependent upon latitude ([23](#_ENREF_23)). Fall migration is slower and individuals depart breeding grounds between mid-August to mid-September. Individuals show very little variation in arrival and departure dates, though variation in dates across a single population is approximately 30 days ([32](#_ENREF_32)). Overall, wood thrushes arrive 6 days earlier than they did 80 years ago ([33](#_ENREF_33)). Arrival dates have been linked to temperature, where wood thrushes arrive earlier in warmer years ([34](#_ENREF_34)).

Because wood thrushes are long distance migrants they may be less adaptable to changes in climate ([5](#_ENREF_5)). This is because environmental indicators on the wintering grounds could be affected differently by climate change than those on the breeding grounds. While recent research shows that individual wood thrushes are not capable of adapting to changing environmental conditions, there may be enough variation in arrival dates across the population (30 day window) to provide some ability to adapt to a change in climate ([32](#_ENREF_32)). CCVI (C.6) was scored “Increase”.

**Modeled Response of Range Distribution**

**Modeled future (2050) change in population or range size (D.2)**

For the 2041-2070 time horizon the CC-QBD modeling framework predicts a range expansion with an increase in habitat area by 25.5%, and a northward range expansion of 28 km/decade for an overall range extension by 304 km ([16](#_ENREF_16)). For the 2071-2100 time horizon the model predicted a continued range expansion, but at a slower rate; northern range limits increased by an additional 119 km and habitat area increased by an additional 11.6% relative to the first time horizon. Overall this resulted in a net increase of 40% of habitat area relative to the 1961-1990 modeled distribution ([16](#_ENREF_16)). However, the deciduous trees that wood thrushes select for nesting are unlikely to move northward as quickly. The northern range limit lies on the boreal shield, where nesting tree species such as American elm, American beech, and red maple are rare.

The US-specific CC-TABA models predicted a decrease in abundance in the western and southern parts of the wood thrush range ([17](#_ENREF_17)). The top predictors in this model were red maple distribution, annual precipitation, American beech distribution, American elm distribution, and the mean difference between July and January temperatures ([18](#_ENREF_18)). Given the northern extent of the modeling, it is difficult to extrapolate the changes at the northern range limit; however, current research suggests that American beech may decline throughout its range across the eastern United States, while American elm may increase within the northern extent of its range across the northern United States ([35](#_ENREF_35)). Tree species migration is generally a slow process, occurring at a rate of <10 km per century, so the northward range expansion of these species could be minimal.

The climate-vegetation modeling predicted a decline in wood thrush incidence in the southern portion of their range, but no decline in the northern part of the US range ([18](#_ENREF_18)). This could mean that northern habitats will be more important for wood thrush conservation, however, it is unknown whether these habitats will expand northwards with the climatic envelope. CCVI (D.2) was scored “Increase”.

**Additional conservation concerns (not directly related to climate change)**

Wood thrushes are a mature forest specialist preferring forests with dense understory. This type of habitat is relatively limited in southern Ontario, where the majority of current wood thrush observations are located provincially. Much of the existing forest in southern Ontario is largely fragmented ([36](#_ENREF_36)), which is detrimental to this area sensitive species because of higher rates of cowbird nest parasitism ([37](#_ENREF_37), [38](#_ENREF_38)). Additionally, adult survivorship is dependent on landscape connectivity; survival rates are 0.58 on fragmented landscapes compared to 0.80 on continuous landscapes ([37](#_ENREF_37)).

Ontario contains approximately 78% of the Canadian wood thrush population ([39](#_ENREF_39)). Wood thrushes have declined 2.1% annually across their range since 1966. Within bird conservation areas, wood thrushes have declined by 1.63% and 3.14% annually since 1966 in the Great Lakes-St. Lawrence region and the Boreal Hardwood Transition region, respectively ([20](#_ENREF_20)). A recent analysis suggests that the wood thrush population in Ontario is experiencing both short- and long-term declines resulting in an overall population loss of 36% in the last 10 years([39](#_ENREF_39)). Wood thrushes are also declining in the United States at approximately 1.9% per year, reducing the potential for a rescue effect for Canadian populations ([39](#_ENREF_39)). Overall, the population size is estimated to be between 250,000-650,000 individuals in Canada ([39](#_ENREF_39)), with the Ontario population estimated at 200,000 wood thrushes ([3](#_ENREF_3)). Additionally, while there is some population growth in southern Ontario, there is an annual decline (-1.6% or less) at the northern range limit within the Great Lakes St. Lawrence ecoregion. This could indicate that this species’ capacity for northward range expansion may be limited, especially given that areas with a wood thrush breeding presence in the Ontario Breeding Bird Atlas in 1981-1985 reported an absence in the second atlas (2001-2005) ([3](#_ENREF_3)). This species is listed as ‘threatened’ nationally and ‘special concern’ in Ontario ([22](#_ENREF_22)).

# Hooded Warbler

**Exposure to Climate Change**

**Expected climate change in breeding and overwintering ranges (A)**

Hooded warblers (*Setophaga citrina*) breed from southern Ontario east to Rhode Island, south through northern Florida and across the Gulf Coast through northeastern Texas ([40](#_ENREF_40)). Within the Great Lakes, hooded warblers are found only in the basins of Lake Michigan, Lake Erie, and Lake Ontario. The wintering range extends from southern Mexico along the Atlantic coast through western Panama and also includes Cuba, Puerto Rico, and the British Virgin Islands ([40](#_ENREF_40)). Within the portion of the hooded warbler breeding grounds contained within the Great Lakes Basin, by 2050, the average temperature is generally expected to rise between 2.2 – 2.7°C. Drought conditions will increase, with an AET:PET moisture index ranging from -0.051 – 0.096. Within the Mexican and Central American overwintering grounds, the CCEI index is largely > 7, indicating exposure to both elevated temperature and drought conditions.

**Sensitivity and Adaptive Capability**

**Dependence on other species to generate required habitat (C.4.a)**

Hooded warblers occupy deciduous forest stands dominated by maple, American beech, and oak ([40](#_ENREF_40)). These birds breed in mature forest that has gaps or openings where early successional vegetation grows. This species will colonize forest stands within 1-2 years after harvest and silvicultural activity ([41](#_ENREF_41)). Hooded warblers will use these shrubby openings for 10-12 years, until the stand reaches a height of 5 m and begins to shade out the undergrowth ([42](#_ENREF_42)). This species utilizes several tree species as habitat, not all of which will be affected strongly by climate change, so we scored CCVI (C.4.a) as “Somewhat Increase”.

**Dietary versatility (C.4.b)**

Hooded warblers are insectivorous, preferring to feed in edge habitat within logged forests ([43](#_ENREF_43)). In the breeding season, hooded warbler foraging occurs mostly at heights of 10-18 m and their summer diet consists largely of small spiders and insects, including Lepidoptera, Coleoptera, Diptera, and Orthoptera ([44](#_ENREF_44), [45](#_ENREF_45)). Hooded warbler could likely adapt to different food sources, so we scored CCVI (C.4.b) as “Neutral”.

**Sensitivity to pathogens or natural enemies (C.4.e)**

A recent study in Ontario examined the impacts of cowbird parasitism on hooded warblers. Of 157 nests initiated, 36% survived; of those that failed, 81% were due to depredation and 16% were due to cowbird parasitism ([46](#_ENREF_46)). Cowbird parasitism was higher in harvested sites, and reference sites were more productive than harvested sites. Overall, fledgling survival is low (0.69-0.72) in the first few days ([40](#_ENREF_40)), however, the number of successfully fledged young is higher in larger patches (>150 ha) than smaller fragments (5-30 ha), due to lower parasitism rates in the larger forest stands ([47](#_ENREF_47)).

One of the nesting tree species for wood thrush is American beech. Beech trees are susceptible to Beech Bark Disease (BBD) ([29](#_ENREF_29)). Because of the relationship between climate and both cowbird parasitism and BBD effects on just one of its habitat tree species, we scored CCVI (C.4.e) as “Somewhat Increase”.

**Phenological response (C.6)**

Hooded warblers depart from wintering grounds in early March and arrive on breeding grounds from late April to late May ([40](#_ENREF_40)). They depart from breeding grounds in late July to late September and arrive on wintering grounds from early August to late October ([40](#_ENREF_40)). Loss of synchrony between food production and arrival time indicates some negative phenological responses, so we scored CCVI (C.6) as “Somewhat Increase”.

**Modeled Response of Range Distribution**

**Modeled future (2050) change in population or range size (D.2)**

All three modeling frameworks predicted northern expansion in range for hooded warbler in relation to different climate scenarios. For the 2041-2070 time horizon the CC-QBD models predicted a range expansion with an increase in habitat area by 28.6%, a northward range expansion of 34 km/decade for an overall range expansion of 378 km ([16](#_ENREF_16)). For the 2071-2100 time horizon the models predict a continued range expansion, but at a slower rate; northern range limits increased by an additional 156 km and habitat area increased by an additional 20.4% relative to the first time horizon. Overall, this resulted in a net increase of 43.4% of habitat area relative to the 1961-1990 modeled distribution ([16](#_ENREF_16)).

The CC-TABA model predicts a decrease in abundance in the southern part of the hooded warbler range and an increase in the northern US part of their range through the eastern seaboard as well as northern Minnesota, northern Wisconsin, and Michigan, including the upper peninsula ([18](#_ENREF_18)). The top predictors in this model are loblolly pine (*Pinus taeda*), cucumber tree (*Magnolia acuminata*), summer precipitation, the mean difference between July and January temperatures, and minimum elevation ([18](#_ENREF_18)). Loblolly pine does not exist in Ontario, and cucumber tree has only a small range in southern Ontario, however, given the predicted increase in distribution across the northern and eastern states, continued range expansion in Ontario is possible. Based on these predictions, we scored the effect the projected range changes on vulnerability, CCVI (D.2), as “Somewhat Increase”.

**Additional conservation concerns (not directly related to climate change)**

Currently, the greatest threat to hooded warbler persistence is habitat loss, not climate change ([48](#_ENREF_48), [49](#_ENREF_49)). Hooded warbler is a gap phase species that prefers mature forest with canopy gaps, which is found in small patches across southern Ontario ([48](#_ENREF_48)). These patches are interspersed with highly valued agricultural lands in southern Ontario. Loss of habitat could be mitigated through conservation efforts to increase deciduous forest cover elsewhere on the landscape, or to manage older stands to encourage canopy openings.

Hooded warblers are increasing at rates of 1.5% annually throughout their range and 4.1% annually in the Great Lakes-St. Lawrence region between 1966 and 2012 ([20](#_ENREF_20)). In Canada, hooded warblers occur only in Ontario where the population is small, but increasing. In 1997 there were an estimated 88 territorial males, this increased to 436 territorial males in 2007, representing less than 1% of the global population ([42](#_ENREF_42)). As a result of the increasing population and expanding range, COSEWIC changed the designation of hooded warbler from ‘threatened’ to ‘not at risk’ ([42](#_ENREF_42)). Additionally, there is evidence of recent range expansion in Ontario ([48](#_ENREF_48)). In Ontario the species is listed as ‘special concern’.

S1 Table A. CCVI scores for eastern meadowlark, wood thrush, and hooded warbler in the Great Lakes Basin (GLB).

| Vulnerability Indices | Eastern Meadowlark | Wood Thrush | Hooded Warbler |
| --- | --- | --- | --- |
| **Section A: Exposure to Local Climate Change** |  |  |  |
| Temperature: Severity (% of GLB) |  |  |  |
| >6.0° F warmer | 0 | 0 | 0 |
| 5.6-6.0° F warmer | 0 | 0 | 0 |
| 5.1-5.5 ° F warmer | 10 | 10 | 0 |
| 4.5-5.0 ° F warmer | 80 | 80 | 50 |
| 3.9-4.4 ° F warmer | 10 | 10 | 50 |
| <3.9 ° F warmer | 0 | 0 | 0 |
| Hamon AET:PET Moisture Metric: Severity (% of GLB) |  |  |  |
| <-0.119 | 0 | 0 | 0 |
| -0.097 - -0.119 | 0 | 0 | 0 |
| -0.074 - -0.096 | 20 | 20 | 50 |
| -0.051 - -0.073 | 60 | 55 | 50 |
| -0.028 - -0.050 | 20 | 20 | 0 |
| >-0.028 | 0 | 5 | 0 |
| Migratory Exposure -- Climate Change Exposure Index: Severity (% of GLB) |  |  |  |
| >7 | 85 | 85 | 80 |
| 6-7 | 10 | 10 | 10 |
| 4-5 | 5 | 5 | 10 |
| <4 | 0 | 0 | 0 |
| **Section B: Indirect Exposure to Climate Change** |  |  |  |
| 1) Exposure to sea level rise |  |  |  |
| 2) Distribution relative to barriers |  |  |  |
| a) Natural barriers | Increase |  |  |
| b) Anthropogenic barriers |  |  |  |
| 3) Predicted impact of land use changes resulting from human responses to climate change | Increase |  |  |
| **C. Sensitivity and Adaptive Capacity Factors** |  |  |  |
| 1) Dispersal and movements |  |  |  |
| 2) Predicted sensitivity to temperature and moisture changes |  |  |  |
| a) Predicted sensitivity to changes in temperature |  |  |  |
| i) historical thermal niche |  |  |  |
| ii) physiological thermal niche |  |  |  |
| b) Predicted sensitivity to changes in precipitation, hydrology, or moisture regime |  |  |  |
| i) historical hydrological niche |  |  |  |
| ii) physiological hydrological niche | Increase/ Somewhat Increase |  |  |
| c) Dependence on a specific disturbance regime likely to be impacted by climate change | Increase |  |  |
| d) Dependence on ice, ice-edge, or snow-cover habitats |  |  |  |
| 3) Restriction to uncommon landscape/geological features or derivatives |  |  |  |
| 4) Interspecific interactions |  |  |  |
| a) Dependence on other species to generate required habitat | Increase/ Somewhat Increase | Increase | Somewhat increase |
| b) Dietary versatility (animals only) |  | Increase |  |
| e) Sensitivity to pathogens or natural enemies |  | Increase | Somewhat increase |
| f) Sensitivity to competition from native or non-native species |  |  |  |
| g) Forms part of an interspecific interaction not covered by 5a-f |  |  |  |
| 5) Genetic factors |  |  |  |
| a) Measured genetic variation |  |  |  |
| b) Occurrence of bottlenecks in recent evolutionary history |  |  |  |
| c) Reproductive system |  |  |  |
| 6) Phenological response to changing seasonal temperature and precipitation dynamics | Increase/ Somewhat Increase | Increase | Somewhat Increase |
| **Section D: Documented or Modeled Response to Climate Change** |  |  |  |
| 1) Documented response to recent climate change |  |  |  |
| 2) Modeled future (2050) change in population or range size | Increase | Increase | Somewhat increase |
| 3) Overlap of modeled future (2050) range with current range | Somewhat Increase | Somewhat Increase | Somewhat increase |
| 4) Occurrence of protected areas in modeled future (2050) distribution |  | Neutral |  |
| **Vulnerability to Climate Change Scores** |  |  |  |
| Climate Change Vulnerability Index (CCVI) | Highly Vulnerable | Highly Vulnerable | Less Vulnerable |
| Confidence in Vulnerability Score | Very High | Very High | Very High |
| Climate Exposure in Migratory Range | High | High | High |
| **Conservation Concern** |  |  |  |
| COSEWIC (National - Canada) | Threatened | Threatened | Not at Risk |
| SARA (Ontario) | Threatened | Special Concern | Special Concern |
| NatureServe G-rank (Global) | Secure | Secure | Secure |

References

1. Lanyon WE, Jaster L, Jensen WE. Eastern Meadowlark (*Sturnella magna*). In: Poole A, editor. The Birds of North America Online: Ithaca: Cornell Lab of Ornithology; 2012.

2. Hamon WR. Estimating potential evapotranspiration. Journal of the Hydraulics Division. 1961;ASCE. 87(HY3):107-20.

3. Cadman MD, Sutherland DA, Beck GG, Lepage D, Courturier AR. Atlas of the breeding birds of Ontario, 2001-2005. Toronto, Ontario: Bird Studies Canada, Environment Canada, Ontario Field Ornithologists, Ontario Ministry of Natural Resources, and Ontario Nature; 2007. 706 p.

4. With KA, King AW, Jensen WE. Remaining large grasslands may not be sufficient to prevent grassland bird declines. Biol Conserv. 2008 Dec;141(12):3152-67. PubMed PMID: WOS:000261574900022.

5. Both C, Van Turnhout CAM, Bijlsma RG, Siepel H, Van Strien AJ, Foppen RPB. Avian population consequences of climate change are most severe for long-distance migrants in seasonal habitats. Proc R Soc B. 2010 Apr 22;277(1685):1259-66. PubMed PMID: WOS:000275381900018.

6. Briggs JM, Knapp AK. Interannual variability in primary production in tallgrass prairie - climate, soil-moisture, topographic position, and fire as determinants of aboveground biomass. American Journal of Botany. 1995 Aug;82(8):1024-30. PubMed PMID: WOS:A1995RQ00500009.

7. Feltmate B, Thistlethwaite J. Climate change adaptation: a priorities plan for Canada. Climate Change Adaptation Project (Canada). 2012.

8. McLaughlin ME, Janousek WM, McCarty JP, Wolfenbarger LL. Effects of urbanization on site occupancy and density of grassland birds in tallgrass prairie fragments. Journal of Field Ornithology. 2014 Sep;85(3):258-73. PubMed PMID: WOS:000341503300003.

9. Kershner EL, Walk JW, Warner RE. Breeding-season decisions, renesting, and annual fecundity of female eastern meadowlarks (*Sturnella magna*) in Southeastern Illinois. Auk. 2004 Jul;121(3):796-805. PubMed PMID: WOS:000222806000015.

10. Kershner EL, Walk JW, Warner RE, Haukos D. Postfledging movements and survival of juvenile Eastern Meadowlarks (*Sturnella magna*) in Illinois. The Auk. 2004;121(4):1146-54.

11. Roseberry JL, Klimstra W. The nesting ecology and reproductive performance of the Eastern Meadowlark. The Wilson Bulletin. 1970:243-67.

12. Wiens JA. An approach to the study of ecological relationships among grassland birds. Ornithological Monographs. 1969:1-93.

13. Boyce MS, McDonald LL. Relating populations to habitats using resource selection functions. Trends in Ecol Evol. 1999;14(7):268-72.

14. Wilson WH. Spring arrival dates of migratory breeding birds in Maine: Sensitivity to climate change. The Wilson Journal of Ornithology. 2007;119(4):665-77.

15. Huppop O, Huppop K. North Atlantic Oscillation and timing of spring migration in birds. Proc R Soc B. 2003 Feb;270(1512):233-40. PubMed PMID: WOS:000181064200002.

16. Berteaux D, Blois Sd, Angers J-F, Bonin J, Casajus N, Darveau M, et al. The CC-Bio Project: studying the effects of climate change on Quebec biodiversity. Diversity. 2010;2(11):1181-204.

17. Matthews SN, Iverson LR, Prasad AM, Peters MP. Changes in potential habitat of 147 North American breeding bird species in response to redistribution of trees and climate following predicted climate change. Ecography. 2011 Dec;34(6):933-45. PubMed PMID: WOS:000297738200004.

18. Matthews SN, Iverson LR, Prasad A, Peters MP. A Climate Change Atlas for 147 Bird Species of the Eastern United States [database]. Delaware, Ohio: Northern Research Station, USDA Forest Service; 2007-ongoing.

19. Butcher GS, Niven DK. Combining data from the Christmas Bird Count and the Breeding Bird Survey to determine the continental status and trends of North America birds. National Audubon Society, 2007.

20. Sauer JR, Hines JE, Fallon JE, Pardieck KL, Ziolkowski J, D. J., Link WA. The North American Breeding Bird Survey, results and analysis 1966-2012. 2014;Version 02.19.2014.

21. McCracken JD, Reid RB, Renfrew RB, Frei B, Jalava JV, Cowie A, et al. Recovery strategy for the bobolink (*Dolichonyx oryzivorus*) and the eastern meadowlark (*Sturnella magna*) in Ontario. Peterborough, Ontario: Ontario Ministry of Natural Resources; 2013. p. 88.

22. COSEWIC. COSEWIC assessment and status report on the Eastern Meadowlark (*Sturnella magna)* in Canada. Ottawa, Canada 2011 [March 2nd, 2015]. Available from: [www.sararegistry.gc.ca/default.asp?lang=En&n=CC00D5CB-1](http://www.sararegistry.gc.ca/default.asp?lang=En&n=CC00D5CB-1).

23. Evans M, Gow E, Roth RR, Johnson MS, Underwood TJ. Wood Thrush (*Hylocichla mustelina*). In: Poole A, editor. The Birds of North America Online: Ithaca: Cornell Lab of Ornithology; 2011.

24. Peck GK, James RD. Breeding birds of Ontario: nidiology and distribution: Royal Ontario Museum Toronto; 1987.

25. Bonnot TW, Thompson FR, Millspaugh JJ, Jones-Farrand DT. Landscape-based population viability models demonstrate importance of strategic conservation planning for birds. Biol Conserv. 2013 Sep;165:104-14. PubMed PMID: WOS:000323871700013.

26. Roth RR, Johnson MS, Underwood TJ. Wood thrush: *Hylocichla mustelina*: American Ornithologists' Union; 1996.

27. Robbins CS, Dawson DK, Dowell BA. Habitat area requirements of breeding forest birds of the middle Atlantic states. Wildlife Monographs. 1989:3-34.

28. Holmes RT, Robinson SK. Spatial patterns, foraging tactics, and diets of ground-foraging birds in a northern hardwoods forest. The Wilson Bulletin. 1988:377-94.

29. Dukes JS, Pontius J, Orwig D, Garnas JR, Rodgers VL, Brazee N, et al. Responses of insect pests, pathogens, and invasive plant species to climate change in the forests of northeastern North America: What can we predict? Canadian Journal of Forest Research. 2009;39(2):231-48.

30. McLaughlin J, Greifenhagen S. A primer and management recommendations. Ontario, Canada: Queen's Printer for Ontario, 2012.

31. Stutchbury BJM, Tarof SA, Done T, Gow E, Kramer PM, Tautin J, et al. Tracking long-distance songbird migration by using geolocators. Science. 2009;323(5916):896-. PubMed PMID: WOS:000263295400031.

32. Stanley CQ, MacPherson M, Fraser KC, McKinnon EA, Stutchbury BJM. Repeat tracking of individual songbirds reveals consistent migration timing but flexibility in route. Plos One. 2012;7(7). PubMed PMID: WOS:000306806600023.

33. Butler CJ. The disproportionate effect of global warming on the arrival dates of short-distance migratory birds in North America. Ibis. 2003;145(3):484-95.

34. Marra PP, Francis CM, Mulvihill RS, Moore FR. The influence of climate on the timing and rate of spring bird migration. Oecologia. 2005 Jan;142(2):307-15. PubMed PMID: WOS:000226285000016.

35. Lele SR, Merrill EH, Keim J, Boyce MS. Selection, use, choice and occupancy: clarifying concepts in resource selection studies. Journal of Animal Ecology. 2013;82(6):1183-91.

36. Ontario Ministry of Natural Resources and Forestry. State of Ontario's forests 2014 [March 2nd, 2015]. Available from: https://[www.ontario.ca/document/state-ontarios-forests](http://www.ontario.ca/document/state-ontarios-forests).

37. Donovan TM, Thompson FR, Faaborg J, Probst JR. Reproductive success of migratory birds in habitat sources and sinks. Conserv Biol. 1995;9(6):1380-95. PubMed PMID: WOS:A1995TL67200008.

38. Etterson MA, Greenberg R, Hollenhorst T. Landscape and regional context differentially affect nest parasitism and nest predation for Wood Thrush in central Virginia, USA. Condor. 2014 May;116(2):205-14. PubMed PMID: WOS:000338095200006.

39. COSEWIC. COSEWIC assessment and status report on the Wood Thrush (*Hylocichla mustelina*) in Canada. 2012 (March 2nd, 2015).

40. Chiver I, Ogden LJ, Stutchbury BJM. Hooded warbler (*Setophaga citrina*). In: Poole A, editor. The Birds of North America Online: Ithaca: Cornell Lab of Ornithology; 2011.

41. Morris DL, Porneluzi PA, Haslerig J, Clawson RL, Faaborg J. Results of 20 years of experimental forest management on breeding birds in Ozark forests of Missouri, USA. For Ecol Manage. 2013 Dec;310:747-60. PubMed PMID: WOS:000330601000078.

42. COSEWIC. COSEWIC assessment and status report on the Hooded Warbler (*Setophaga citrina*) in Canada Ottawa, Canada2012 [March 2nd, 2015]. Available from: [www.sararegistry.gc.ca/default.asp?lang=En&n=1502E054-1](http://www.sararegistry.gc.ca/default.asp?lang=En&n=1502E054-1).

43. Bowen LT, Moorman CE, Kilgo JC. Seasonal bird use of canopy gaps in a bottomland forest. The Wilson Journal of Ornithology. 2007;119(1):77-88.

44. Bent AC. Life histories of North American wood warblers. U.S. National Museum, 1953.

45. Moorman CE, Bowen LT, Kilgo JC, Sorenson CE, Hanula JL, Horn S, et al. Seasonal diets of insectivorous birds using canopy gaps in a bottomland forest. Journal of Field Ornithology. 2007 Win;78(1):11-20. PubMed PMID: WOS:000244553500002.

46. Eng ML, Stutchbury BJM, Burke DM, Elliott KA. Influence of forest management on pre- and post-fledging productivity of a Neotropical migratory songbird in a highly fragmented landscape. Canadian Journal of Forest Research. 2011 Oct;41(10):2009-19. PubMed PMID: WOS:000297275500011.

47. Rush SA, Stutchbury BJM. Survival of fledgling Hooded Warblers (*Wilsonia citrina*) in small and large forest fragments. Auk. 2008 Jan;125(1):183-91. PubMed PMID: WOS:000256372500021.

48. Melles SJ, Fortin MJ, Lindsay K, Badzinski D. Expanding northward: influence of climate change, forest connectivity, and population processes on a threatened species' range shift. Glob Change Biol. 2011 Jan;17(1):17-31. PubMed PMID: WOS:000284851500002.

49. Naujokaitis-Lewis IR, Curtis JMR, Tischendorf L, Badzinski D, Lindsay K, Fortin MJ. Uncertainties in coupled species distribution-metapopulation dynamics models for risk assessments under climate change. Divers Distrib. 2013 May-Jun;19(5-6):541-54. PubMed PMID: WOS:000318507800006.
